# Bacterial and Fungal Communities Are Differentially Modified by Melatonin in Agricultural Soils Under Abiotic Stress

**DOI:** 10.3389/fmicb.2019.02616

**Published:** 2019-12-03

**Authors:** Andrew P. Madigan, Eleonora Egidi, Frank Bedon, Ashley E. Franks, Kim M. Plummer

**Affiliations:** ^1^Department of Animal, Plant and Soil Sciences, AgriBio, La Trobe University, Melbourne, VIC, Australia; ^2^Department of Physiology, Anatomy and Microbiology, La Trobe University, Melbourne, VIC, Australia; ^3^Hawkesbury Institute for the Environment, Western Sydney University, Richmond, NSW, Australia; ^4^Centre for Future Landscapes, School of Life Sciences, La Trobe University, Melbourne, VIC, Australia

**Keywords:** melatonin, microbial ecology, automated ribosomal intergenic spacer analysis, abiotic stress, salt, cadmium

## Abstract

An extensive body of evidence from the last decade has indicated that melatonin enhances plant resistance to a range of biotic and abiotic stressors. This has led to an interest in the application of melatonin in agriculture to reduce negative physiological effects from environmental stresses that affect yield and crop quality. However, there are no reports regarding the effects of melatonin on soil microbial communities under abiotic stress, despite the importance of microbes for plant root health and function. Three agricultural soils associated with different land usage histories (pasture, canola or wheat) were placed under abiotic stress by cadmium (100 or 280 mg kg^−1^ soil) or salt (4 or 7 g kg^−1^ soil) and treated with melatonin (0.2 and 4 mg kg^−1^ soil). Automated Ribosomal Intergenic Spacer Analysis (ARISA) was used to generate Operational Taxonomic Units (OTU) for microbial community analysis in each soil. Significant differences in richness (α diversity) and community structures (β diversity) were observed between bacterial and fungal assemblages across all three soils, demonstrating the effect of melatonin on soil microbial communities under abiotic stress. The analysis also indicated that the microbial response to melatonin is governed by the type of soil and history. The effects of melatonin on soil microbes need to be regarded in potential future agricultural applications.

## Introduction

Soil microbial communities have an essential role in maintaining ecosystem health. Microbes can exchange nutrients and minerals directly with the plant root systems as well as indirectly benefit plant growth through nutrient cycling of organic matter in soils (Wall and Virginia, 1999; Yao et al., 2000; Kirk et al., 2004; Wahid et al., 2016). The diversity and structure of microbial communities in soils can be altered by various abiotic stresses. Community alterations can have important consequences for ecosystem-level functions provided by soil microbes (Mau et al., 2019; Zhang et al., 2019). Among the various abiotic stresses that pose a significant threat to many terrestrial systems, increasing levels of salinity and contaminants due to anthropogenic activities have a prominent role (Badri and Vivanco, 2009; Wood et al., 2016a,b; Geisseler et al., 2017).

Mining, industrial air pollution and phosphorus fertilizer treatments have been responsible for introducing vast quantities of the highly toxic heavy metal cadmium into the environment (Jiao et al., 2012; Khairy et al., 2014; Qadir et al., 2014). In China alone, 743 metric tons of cadmium were released by such anthropogenic activities in a single year (Cheng et al., 2014), causing cadmium to be the most abundant heavy metal contaminant in agricultural soils (Wang et al., 2019). Cadmium is a serious health issue in humans due to its ability to accumulate in edible crops such as rice (*Oryza sativa*) and soybean (*Glycine max*) (Franzaring et al., 2019; Wang et al., 2019). Contaminated irrigation water is a common source of cadmium or salt into agricultural soils (Khairy et al., 2014; Qadir et al., 2014; Roberts, 2014; Bencherif et al., 2015; Franzaring et al., 2019). Up to 2 million hectares of agricultural land is negatively impacted by salinization each year globally (Bencherif et al., 2015; Ke et al., 2017). Salt stress can impact ion homeostasis, photosynthetic capacity, and root and shoot dry weight in plants (Zhan et al., 2019).

In microbes, both cadmium toxicity and soil salinization cause a dramatic increase in cellular levels of highly destructive reactive oxygen species (ROS) as well as inhibit the activities of ROS scavenging enzymes (Tanaka et al., 2006; Achard-Joris et al., 2007; Hossain et al., 2012). Soil microbial community structures and activities are altered by cadmium toxicity and salinity (Cáliz et al., 2013; Rath et al., 2016; Wood et al., 2016a,b; Thiem et al., 2018). Such alterations to the soil microbial community have important repercussions in agricultural settings, where microbes (both soil bacteria and fungi) are critical drivers of soil health and agricultural crop productivity (Mau et al., 2019; Zhang et al., 2019). As such, exploring novel avenues to control stress-induced shifts in soil microbial community composition and functionality could be a key to unfolding agricultural constraints and achieve better agricultural productivities and sustainable development in the future.

Melatonin (N-acetly-5-methoxytryptamine) is an indoleamine (secondary metabolite) produced by all cellular organisms (Hardeland, 2015; Manchester et al., 2015). In plants, melatonin has been shown to decrease the physiological deleterious effect of various abiotic stresses (Zhang et al., 2015; Nawaz et al., 2016; Sharif et al., 2018; Debnath et al., 2019). Exogenous melatonin application *via* seed-coatings (Wei et al., 2015), soil treatments (Cui et al., 2017), or foliar sprays (Zhang et al., 2017a,b) have been described to promote growth and protect plants against stressors such as cadmium (Byeon et al., 2015; Li et al., 2016; Gu et al., 2017) and salt (Li et al., 2012; Liang et al., 2015; Jiang et al., 2016). Melatonin can act either as a highly efficient antioxidant, scavenging up to 10 ROS per molecule, or as a signaling molecule regulating enzymes or hormones associated with ROS scavenging (Weeda et al., 2014; Reiter et al., 2015, 2016; Zhang et al., 2015; Hardeland, 2016; Nawaz et al., 2018). Melatonin exposure alters microbiota composition in the gut (Schultz et al., 2006; Chen et al., 2011) and may have antibiotic activity against some microbes (Lucchelli et al., 1997). However, very little is known about the effects of this secondary metabolite on individual microbes or mixed microbial communities in soil, especially under abiotic stress conditions (Tan et al., 2014; Manchester et al., 2015; Paulose et al., 2016). Therefore, the viability of melatonin application to future agricultural practices may be explored by investigating how melatonin influences soil microbial community dynamics.

The aim of this research is to advance our understanding of how chemically stressed soil microbial communities respond to exogenous melatonin, thus enhancing knowledge of potential plant-soil microbe interactions under the presence of this secondary metabolite. We examined the effects of melatonin on microbial community structures in three different agricultural soils which were artificially stressed with cadmium or salt. This study is focused upon answering: How does melatonin affect the α and β diversity of soil microbes? How similar are the responses of bacterial and fungal communities to melatonin and/or abiotic stresses? Are microbial community responses to melatonin and/or abiotic stresses similar across the different agricultural soils? We hypothesized that: (1) bacteria would be more responsive to treatments compared to fungi; (2) melatonin would impact microbial community structures in soils (a) unstressed and (b) under abiotically stressed conditions to varying degrees across the three soils; (3) specific OTUs would be particularly responsive under the various conditions. This is the first study to date to report significant responses of microbes to melatonin under abiotic stress in agricultural soils at a community level.

## Materials and Methods

### Soils Origin, Sampling, and Physiochemical Characteristics

Three agricultural soils associated with different land uses (pasture and crops) were collected from sites within Victoria, Australia. Prior to sampling in late March and early April 2017, site “P” (37^°^32′28.1″ S, 145^°^05′42.5″ E) was most recently (<3 months) associated with cattle and sheep Pasture, site “C” (37°45′28.4″ S, 144°14′30.2″ E) was 3 weeks post Canola harvest, and site “W” (37°43′31.1″ S, 144°13′14.3″ E) was 3 weeks post-fire blazing of stubble following a Wheat harvest. As the plant species associated with a soil has been shown to be a key factor influencing the bacterial community structure within the rhizosphere (Burns et al., 2015), it was expected that selecting these soils would provide the most diverse range of soil microbes for the current study.

At each site, approximately 4 kg topsoil was sampled (10 cm deep × 0.5 m width × 0.5 length) from each of four plots spaced 3 m apart. Air temperature ranged approximately 28°C/12°C day/night at the time of sampling and soils were dry for at least a week when sampled in the field. Soil samples were subsequently air dried overnight and sieved to remove particles larger than 2 mm. A single soil sample was generated for each site by pooling all four collected soils from the subsite plots (Aye et al., 2016; Butterly et al., 2016). The collected stock soils were separately stored at ambient room temperature (21°C) in airtight plastic containers for 4 months, followed by subsampling (representing the first sampling timepoint; labeled as “T0” in the analysis) for immediate treatments as described below. During this first soil subsampling process stock soils were thoroughly aerated. The stock soils were then stored at ambient room temperature (21°C) in airtight plastic containers for a further 4 weeks and subsampled for immediate treatments (representing the second sampling timepoint; labeled as “T1” in the analysis). It was expected that this aging regime would change the initial microbial populations and potentially shift selection pressures among the populations (Kelly et al., 1999; Castro et al., 2010; Heijboer et al., 2018; Reese et al., 2018). The four-week lag between T0 and T1 sampling timepoints was introduced to reduce any effects on soil microbial community structures within soils caused by the disturbance of soils associated with subsampling. Physicochemical analyses of untreated soils were conducted by Nutrient Advantage (Melbourne, Australia) (Table 1). Electrical conductivities (EC) of untreated and salt-treated (NaCl) soils were determined using 1:5 soil:water as per He et al. (2012) (Table 2).

**Table 1:** Physical and chemical characteristics of the three agricultural soils used in this study. Four topsoil samples (0–10 cm) were collected at each site and composited prior to analysis.

Site	P	C	W
Available potassium (mg kg^−1^ soil)	640	140	170
pH (1:5 CaCl_2_)	6.1	4.9	4.7
Organic carbon (%)	4.6	3	2.8
Nitrate N (mg kg^−1^ soil)	160	57	34
Ammonium N (mg kg^−1^ soil)	2.5	8.9	8.8
Phosphorus (Colwell) (mg kg^−1^ soil)	160	41	32
Phosphorus buffer index	32	71	83
Calcium [cmol(+) kg^−1^ soil]	12	5.1	4.7
CEC [cmol(+) kg^−1^ soil]	15.7	7.94	8.56
EC (1:5 water) (dS m^−1^)	0.37	0.24	0.18
Chloride (mg kg^−1^ soil)	37	67	76
Moisture (%)	14	6	5.2
Water holding capacity (%)	38.4	24.6	21.1
Sand (%)	75.2	53.9	52.4
Silt (%)	23.6	29.9	28.8
Clay (%)	1.2	16.2	18.8
Iron (mg kg^−1^ soil)	5,400	16,000	21,000
Zinc (mg kg^−1^ soil)	23	7.2	9.3
Cadmium (mg kg^−1^ soil)	0.24	0.13	0.2
Chromium (mg kg^−1^ soil)	11	17	32
Nickel (mg kg^−1^ soil)	4	4.7	22
Lead (mg kg^−1^ soil)	7.7	9.4	16

*CEC, cation exchange capacity; EC, electrical conductivity; Soil P, recently (<3 months) associated with cattle and sheep pasture; Soil C, sampled 3 weeks post canola harvest; Soil W, sampled 3 weeks post-fire blazing following wheat harvest*.

**Table 2:** Electrical conductivity (milli Siemens/centimeter) (mS/cm) in soils P, C and W treated with high (7 g kg^−1^ soil) and low (4 g kg^−1^ soil) salt (NaCl).

EC 1:5 (mS/cm)									
	Soil P			Soil C			Soil W		
	7 g kg^−1^	4 g kg^−1^	Control	7 g kg^−1^	4 g kg^−1^	Control	7 g kg^−1^	4 g kg^−1^	Control
Rep 1	2.6	1.72	0.706	1.97	1.015	0.091	1.934	1.148	0.101
Rep 2	2.545	1.708	0.678	1.943	1.083	0.132	1.954	1.089	0.093
Rep 3	2.502	1.8	0.681	1.829	1.084	0.138	1.983	1.065	0.102
Mean	2.549	1.743	0.688	1.914	1.061	0.120	1.957	1.101	0.099
SE	0.028	0.029	0.009	0.043	0.023	0.015	0.014	0.025	0.003

*Control treatments consist of sterile Milli-Q water replacing salt solutions. All treatments and controls within the same soil were significantly different (*p <* 0.05) from each other as determined by one-way ANOVA. Salinity levels as measured by electrical conductivity [EC (1:5 soil:water)]. SE, standard error*.

### Soils Treatment With Abiotic Stress (Cadmium or Salt) and Melatonin

The responses of soil microbial communities to exogenous melatonin application were investigated with and without cadmium and salt as separately applied stressors (all chemicals from Sigma Aldrich Pty. Ltd., Australia). Five grams of sieved topsoil was transferred to a sterile 50 ml Falcon tube and exposed to various treatment combinations. At sampling timepoint T0, treatments were composed of high or low melatonin (4 or 0.2 mg kg^−1^ soil, respectively) and/or high or low cadmium (cadmium chloride hemipentahydrate) (280 or 100 mg kg^−1^ soil, respectively). At sampling timepoint T1, dry (untreated) soils were treated with a solution composed of melatonin (4 or 0.2 mg kg^−1^ soil) and/or high or low salt (NaCl) stressor (7 or 4 g kg^−1^ soil, respectively). Controls involved dilute ethanol replacing melatonin (see below) and/or sterile Milli-Q water replacing cadmium. These concentrations were selected to be within the range of those reported to induce effects on soil microbial activities in previous studies for cadmium (Cáliz et al., 2013; Wood et al., 2016a,b) and salt (Rath et al., 2016). Based upon preliminary studies, soils were treated to 80–90% field capacity, to ensure all soils were sufficiently exposed to stressor and melatonin. Soils without the addition of a stressor acted as a control. Treatments and controls were conducted in quadruplicates. Samples were incubated in sterile 50 ml falcon tubes covered with loosely fitted lids to enable gas exchange at room temperature (21°C) in darkness for 10 days. Four untreated samples (i.e., no water added) replicates per soil were also collected on Day 0 to provide a representation of the baseline communities prior to treatments.

Melatonin was initially dissolved in 99.9% ethanol to 200 mM and diluted to the required concentrations with sterile Milli-Q water. All treatments and controls contained a standardized amount of ethanol (100 μl of 0.43% ethanol per 5 g soil). Due to differing water holding capacities for each soil, this equated to a final dilute ethanol concentration of 0.044, 0.052, and 0.06% v/v in treatments within soils P, C, and W respectively. Similarly, high melatonin (4 mg kg^−1^ soil) treatments corresponded to equivalent concentrations of 88.3, 103.1, and 120.3 μM melatonin, respectively, while low melatonin (0.2 mg kg^−1^ soil) corresponded to equivalent concentrations of 4.4, 5.2, and 6.0 μM melatonin respectively. Control treatments were composed of dilute ethanol replacing melatonin and sterile Milli-Q water replacing cadmium (sampling timepoint T0) or NaCl (sampling timepoint T1).

### DNA Extraction and Amplification for Automated Ribosomal Intergenic Spacer Analysis

Automated Ribosomal Intergenic Spacer Analysis (ARISA) is a molecular technique used to characterize the microbial diversity within various environments, including bulk soil and the rhizosphere (Sørensen et al., 2009; Rincon-Florez et al., 2013). This technique fingerprints fungal and bacterial communities based upon the length heterogeneity of the intergenic spacer region between the ribosomal ribonucleic acid (rRNA) genes for bacteria (16S and 23S) and/or fungi (18S and 28S) (Kirk et al., 2004). ARISA provided a valuable estimate of both species richness and relative abundances, allowing a comparison of α and β diversity both across various sites and between samples exposed to different treatments (Zancarini et al., 2012).

Total soil DNA was extracted from 0.3 g of soil subsamples using PowerSoil^®^ DNA Isolation Kit (MoBio Laboratories Inc., California, USA) according to manufacturer’s instructions. For the fungal community analysis, the internal transcribed spacer regions 1 and 2 (ITS 1 and 2) were amplified using fungal primers ITS-1F and ITS-4 (Blaalid et al., 2013). A 20 μl fungal ARISA PCR mastermix contained 1× PCR buffer (Qiagen Pty Ltd., Melbourne, Australia); 1.5 mM MgCl_2_ (Qiagen); 1× Q reagent (Qiagen); 500 μM concentration of each deoxynucleoside triphosphate (dNTP) (Qiagen); 10 ng of extracted DNA; 500 nM of fungal primers ITS 1F (5′-CTT GGT CAT TTA GAG GAA GTA-3′) and ITS 4 (5′-TCC TCC GCT TAT TGA TAT GC-3′) (Bioline Global Pty Ltd., NSW, Australia), the latter labeled with a phosphoramidite dye, 6FAM (Sigma Aldrich Pty Ltd., Sydney, Australia); and 3.75 U of GoTaq polymerase (Qiagen). Reaction mixtures were held at 96°C for 3 min, followed by 36 cycles of amplification at 96°C for 30 s, 55°C for 75 s, and 72°C for 90 s and a final extension of 72°C for 6 min using a CFX Connect Real-Time PCR Detection System (Bio-Rad, Hercules, USA).

Bacterial community analysis by ARISA targeted the intergenic spacer region of the 16S – 23S rRNA genes using the universal primer 16S – 1392f (5′ – GSA CAC ACC GCC CGT – 3′), labeled with a phosphoramidite dye, 6FAM, and bacterial primer 23S – 125r (5′ – GGG TTB CCC CAT TCR G – 3′) (Fuhrman et al., 2008). The bacterial ARISA PCR mixture contained the same ingredients and concentrations as applied in the fungal PCR mastermix (described above), with the exception of the primers used. Reaction mixtures were held at 94°C for 3 min, followed by 33 cycles of amplification at 94°C for 1 min, 52°C for 1 min, and 72°C for 90 s and a final extension of 72°C for 6 min using a CFX Connect Real-Time PCR Detection System (Bio-Rad, Hercules, USA). PCR products were examined by gel electrophoresis on a 1.5% agarose gel matrix and imaged by fluorescence under UV light. Bacterial and fungal community analyses by ARISA were conducted by Australian Genome Research Facility as per Wood et al. (2016a,b). This involved the separation of amplicons, described as operation taxonomic units (OTUs), by capillary electrophoresis.

### Statistical Analysis of Automated Ribosomal Intergenic Spacer Analysis Data

OTU fragment sizes were limited to a range of 140–1,000 bp for both fungi and bacteria to ensure only the intergenic spacer region was represented in the data (Fisher and Triplett, 1999). Singletons and low abundance amplicons (<1% relative abundance) were disregarded (Fisher and Triplett, 1999). Data were normalized in the statistical software R version 3.3.2 (The R Foundation for Statistical Computing, Boston, USA), with bin sizes of 3 and 4 selected for bacteria and fungi, respectively (Ramette, 2009; Butterly et al., 2016). The following analyses were conducted in Primer-E v6 (Quest Research Ltd., Auckland, New Zealand), with treatments considered as fixed factors and microbial responses analyzed for each soil separately (Chow et al., 2013; Wood et al., 2016a,b): (1) Bray-Curtis similarity for microbial communities; (2) SIMPER analysis to identify the contribution of individual Operational Taxonomic Units (OTUs) to (dis)similarity between replicates or different treatments; (3) DIVERSE to determine Shannon diversity index (*H*′) for sample data; (4) permutational multivariate analysis of variance (PERMANOVA) to determine treatment effect (melatonin, stressors: cadmium and salt) on microbial assemblages for each soil at various concentrations (high, low, and zero). Monte Carlo statistical analysis was conducted to determine if individual treatment combinations had statistically significant effects on community compositions (Van Wijngaarden et al., 1995). Shannon’s diversity index (*H*′) was calculated from binned data to determine differences in biodiversity (relative abundance and evenness) of taxa present within each soil for fungal and bacterial communities upon treatment with melatonin (averaged for four replicates) (Ondreičková et al., 2016). Subsequently, Shannon index values were analyzed by non-parametric Wilcoxon test to determine significant (*p <* 0.05) differences upon melatonin treatments under high or low concentration for the stressors (cadmium or Salt). Non-multidimensional scaling (nMDS) and principal coordinates analyses (PCoA) plots were generated using ggplot and phyloseq packages in the statistical software R v 3.5.2 (The R Foundation for Statistical Computing, Boston, USA) for visual representation of community (dis)similarities between samples.

### Microbial Biomass Response to Melatonin and Stressors

Bacterial and fungal biomasses were measured by quantitative PCR (qPCR) on DNA of the 16 s rRNA and ITS region, respectively. Bacterial communities were assessed using primer pairs 1114f (5′-CGG CAA CGA GCG CAA CCC-3′) -1275r (5′-CCA TTG TAG CAC GTG TGT AGC C-3′), and fungal communities were assessed using ITS1F (5′-TCC GTA GGT GAA CCT GCG G-3′) -5.8Sr (5′-CGC TGC GTT CTT CAT CG-3′) primer (Wood et al., 2016a,b). Each treatment consisted of four biological replicates each quantified in technical triplicate using a CFX Connect Real-Time PCR Detection System (Bio-Rad, Hercules, USA). A 20 μl reaction mixture for bacterial samples was composed of 2 μl extracted DNA (0.25 ng/μl) and 18 μl mastermix according to the following recipe: 3.3 μl Universal SYBR^®^ Green Super Mix; 0.27 μl of each 10 μM forward and reverse primer (135 pM final concentration of each primer in reaction mixture); 14.16 μl DNA-free water. Bacterial qPCR reaction mixtures were held at 94°C for 3 min, followed by 40 cycles of amplification at 94°C for 10 s, 61.5°C for 30 s. Fungal samples were prepared to a 10 μl reaction mixture composed of 2 μl extracted DNA (0.25 ng/μl) and 8 μl mastermix composed of 4.5 μl Universal SYBR^®^ Green Super Mix; 0.5 μl of each 10 μM forward and reverse primer (500 pM final concentration of each primer in reaction mixture); 2.5 μl DNA-free water. Fungal qPCR conditions were 95°C for 5 min, followed by 40 cycles of amplification at 95°C for 30 s, 53°C for 30 s, and 72°C for 30 s. A melting curve was measured from 65°C up to 95°C following qPCR reactions by increasing in 0.5°C increments every 30 s. Purified amplicons from pure isolates of *E. coli* and *Penicillium* sp. cultures were used to generate standard curves (10-fold series dilutions) for bacterial and fungal samples, respectively.

## Results

### Agricultural Soils Have Diverse Microbiomes and Physiochemical Properties

**Figure 1:**
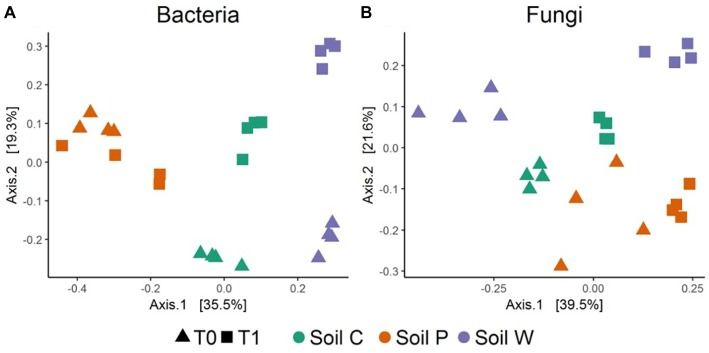
Principal coordinates analysis (PCOA) ordination plots (Bray-Curtis distance matrix) of ARISA profiles showing the separation within **(A)** bacterial and **(B)** fungal communities for the three dry, untreated soils (P, C, W; *n* = 4). Bacterial communities from the same soil differed (ANOSIM: *R* = 0.865–1; *p* = 0.029) between sampling timepoints T0 and T1 within soils C and W, whereas bacterial communities in soil A were not significantly different between timepoints T0 and T1 (ANOSIM: *R* = 0.594; *p* = 0.057). All untreated soil communities from different soils were dissimilar (*p <* 0.05) to each other. Fungal communities from the same soil differed (ANOSIM: *R* = 0.458–1; *p* = 0.029) between T0 and T1 for all three soils.

Three agricultural soils differing in land use practices, physiochemical characteristics, and microbial content were used to assess the modulatory effect of melatonin on soil community. Soils C and W were collected from sites within close geographical proximity (<10 km) to each other and showed the most similarity in physiochemical parameters of the three soils (Table 1). Electrical conductance substantially increased in response to both salt treatments (Table 2). The ARISA community profiles representing bacterial and fungal communities in the three dry untreated soils showed significant (*p <* 0.05) differences to each other (Figure 1). We detected a mean of 29.71, 30.00, and 36.00 bacterial OTUs, and 16.56, 16.38, and 14.75 fungal OTUs, for untreated soils P, C, and W, respectively.

### Cadmium and Salt Alter Microbial Community Structures

Stressors significantly (*p <* 0.05) impacted microbial β diversity (Table 3). Bacterial community structures of soils C and W were substantially impacted (*p <* 0.01) by treatments with high and low concentrations of cadmium and salt (Supplementary Table S1A). Similarly, bacterial community structures in soil P were responsive to salt at high and low concentrations, but community structures in this soil did not show a significant response to cadmium. Fungal communities in all soils were impacted by high concentrations of cadmium and salt. In contrast, low concentrations of salt impacted fungal community structures in soil C and W (not P), whereas only fungal communities in soil P were altered by low cadmium treatment (Supplementary Table S1B).

**Table 3:** Bacterial (1) and fungal (2) community responses to treatments (β diversity) with melatonin, stressors and melatonin-stressor combinations based on PERMANOVA analyses of ARISA data (Bray-Curtis dissimilarity distances).

		MT		Stressor		MT × Stressor	
		Pseudo-F	*p*	Pseudo-F	*p*	Pseudo-F	*p*
**1. Bacterial community response**							
Soil P	Cd (T0)	1.429	0.1412	1.516	0.1121	1.975	0.0195^*^
	Salt (T1)	2.418	0.0006^***^	3.072	0.0002^***^	1.425	0.046^*^
Soil C	Cd (T0)	2.151	0.0213^**^	9.151	0.0001^***^	0.941	0.546
	Salt (T1)	3.166	0.0029^**^	19.587	0.0001^***^	8.721	0.0001^***^
Soil W	Cd (T0)	10.668	0.0001^***^	8.795	0.0001^***^	2.277	0.0001^***^
	Salt (T1)	7.839	0.0001^***^	10.595	0.0001^***^	10.232	0.0001^***^
**2. Fungal community response**							
Soil P	Cd (T0)	1.1462	0.2957	2.8194	0.0008^***^	1.123	0.2854
	Salt (T1)	1.4014	0.0811	1.8788	0.0054^**^	1.414	0.0284^*^
Soil C	Cd (T0)	1.0284	0.4271	2.1996	0.0071^**^	1.2995	0.1264
	Salt (T1)	1.1854	0.2517	4.2529	0.0001^***^	1.553	0.0167^*^
Soil W	Cd (T0)	4.7926	0.0001^***^	1.8998	0.035^*^	0.7398	0.8123
	Salt (T1)	2.1305	0.0062^**^	6.0265	0.0001^***^	1.8435	0.0033^**^

MT, melatonin; Stressors: cadmium (Cd), Salt. Sampling timepoints: T0, T1. All treatments and controls were composed of a standardized amount of dilute ethanol. *n* = 4 replicates per treatment. Significance of PERMANOVA (highlighted in bold): ^*^0.01 < *p* ≤0.05; ^**^0.001 < *p* ≤0.01; ^***^p ≤0.001.

### Melatonin Affects Soil Microbial Diversity and Abundance Across all Three Soils

#### Soil Microbial Community Responses to Melatonin Under Abiotic Stress Conditions

**Figure 2:**
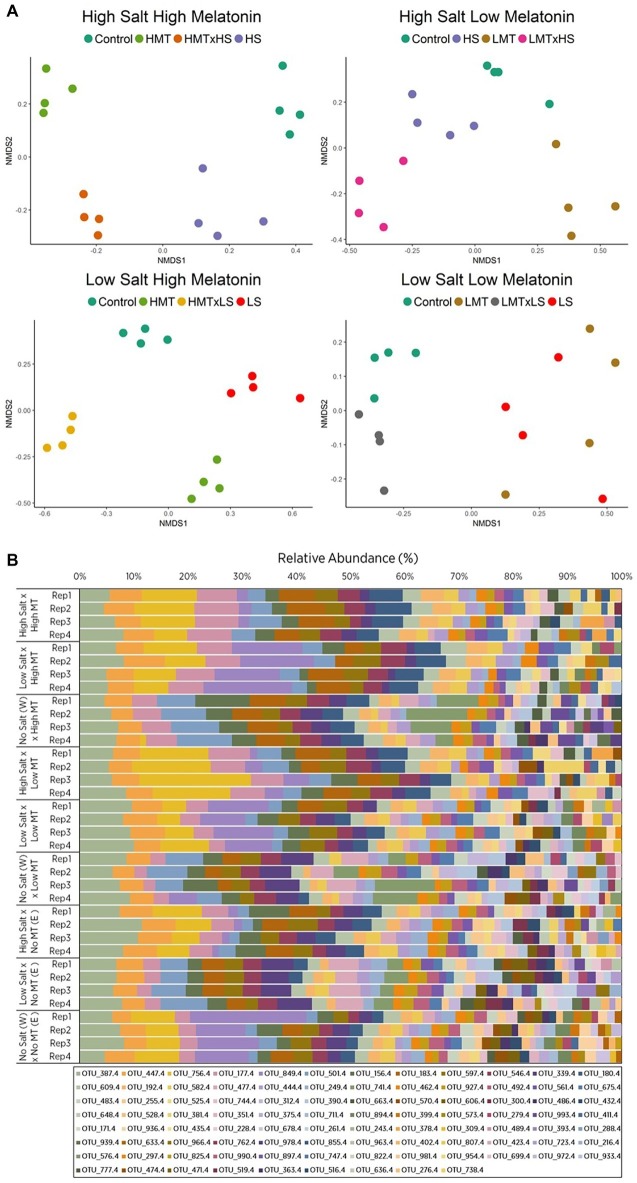
Comparison of **(A)** non-metric multidimensional scaling (nMDS) ordination and **(B)** OTU relative abundances displaying Bray-Curtis similarities for bacterial samples within soil W for various treatments of melatonin and salt based upon community compositions determined by ARISA fingerprinting analysis. Legends in **(B)** represent different individual OTUs as determined by specified nucleotide lengths. The relative proximity of replicates reflects high community similarity within the same treatments for bacterial communities. Water (W) replaced salt treatment and dilute ethanol (E) replaced melatonin treatments in respective control samples. All treatments and controls were composed of a standardized amount of dilute ethanol. HMT, high melatonin; LMT, low melatonin; HS, high salt; LS, low salt; HMT × HS, high melatonin with high salt; HMT × LS, high melatonin with low salt; LMT × HS, low melatonin with high salt; LMT × LS, low melatonin with low salt.

**Figure 3:**
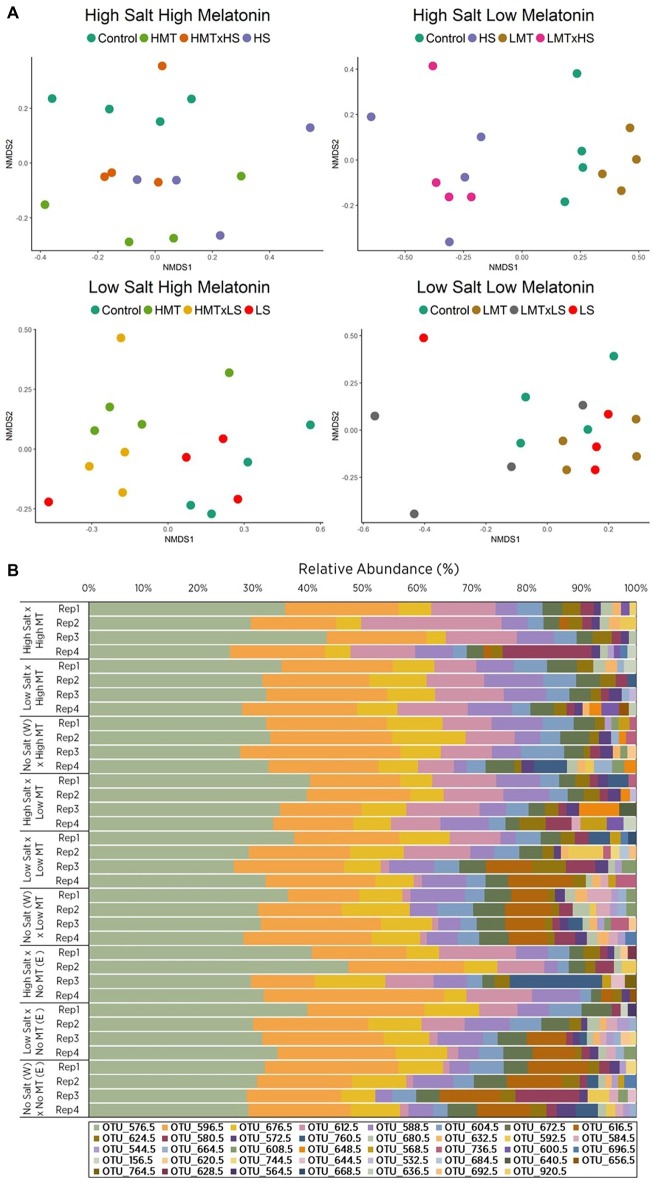
Comparison of **(A)** non-metric multidimensional scaling (nMDS) ordination and **(B)** OTU relative abundances displaying Bray-Curtis similarities for fungal samples within soil W for various treatments of melatonin and salt based upon community compositions determined by ARISA fingerprinting analysis. Legends in **(B)** represent different individual OTUs as determined by specified nucleotide lengths. The relative proximity of replicates reflects high community similarity within the same treatments for fungal communities. Water (W) replaced salt treatment and dilute ethanol (E) replaced melatonin treatments in respective control samples. All treatments and controls were composed of a standardized amount of dilute ethanol. HMT, high melatonin; LMT, low melatonin; HS, high salt; LS, low salt; HMT × HS, high melatonin with high salt; HMT × LS, high melatonin with low salt; LMT × HS, low melatonin with high salt; LMT × LS, low melatonin with low salt.

The addition of melatonin to all three soil types had considerable effects (increases and decreases) on the bacterial α diversity (*p <* 0.05) but did not significantly affect the fungal α diversity (Supplementary Data S1, Supplementary Table S2). Individual bacterial and fungal OTUs that responded strongly to melatonin showed relative abundances increased by up to 7.09 and 11.53% and reduced by 16.92 and 10.23%, respectively (Supplementary Tables S3, S4). Analysis of the soils OTUs compositions (β diversity) by Permutational Multivariate Analysis of Variance (PERMANOVA) indicated that bacterial and fungal community was clearly altered by melatonin and stressors application (Table 3, Supplementary Tables S1, S5, S6). The responses of microbial communities in all soils to melatonin under abiotic stress conditions were visualized by non-metric multidimensional scaling (nMDS) ordination (Figures 2A, 3A, Supplementary Figures S1, S2). Under abiotic stress conditions, bacterial communities responding significantly (*p <* 0.05) to melatonin showed a decreased Shannon diversity index (OTU abundance and evenness), whereas fungal communities increased under the same conditions (Supplementary Data S2, Supplementary Figures S3, S4). Overall melatonin had very little effect on bacterial 16S or fungal 18S rDNA copy numbers, with only one soil bacterial community (Soil W) impacted (*p <* 0.05) by exogenous melatonin only, while fungal communities were unaffected by melatonin-only treatments (Supplementary Data S3, Supplementary Figures S5, S6). Some differences between treatments were recorded, however no consistent pattern of microbial biomass shift was observed under stress conditions +/− melatonin (Supplementary Data S3).

#### Effect of Melatonin on Bacterial and Fungal Communities

Melatonin had different impacts on bacterial versus fungal composition of soils. High melatonin concentration had a large effect (PERMANOVA: *p <* 0.01) on bacterial community structures in all three soils, whereas low melatonin concentration impacted only bacterial communities within soil C and W (Supplementary Table S5A). Fungi responded less to melatonin compared to bacteria within the same samples. Melatonin had no effect on fungal community structures in soil C (Supplementary Table S5B), while only high melatonin concentration treatment at sampling timepoint T1 impacted fungal community structures in soil P (Table 3). However, melatonin at high and low concentration induced shifts in fungal community structures within soil W.

#### Microbial Community Responses to Melatonin Under Stress Condition

In comparison to stressor-only treatments, consistent responses to melatonin were observed in bacterial communities compared to fungal communities, especially at high melatonin concentration (Table 4, Supplementary Data S4, Supplementary Table S6). Bacterial responses to high melatonin concentration were significant (in comparison with stressor only treated communities) under high cadmium or salt conditions in soils P and W, but not in soil C (Table 4). Under low stressor conditions, bacterial communities in soil C and soil W were altered (*p* < 0.05) by high melatonin treatment. In contrast, bacterial communities responded to the availability of low melatonin under high stress conditions only in soil W only (both stressors) and under low stressor (salt only) in soils C and W. Fungal communities were far less responsive to melatonin treatments under abiotic stress conditions. In comparison with stressor only treatments, melatonin only caused a shift in fungal communities at high melatonin treatments in soil W under high cadmium stress and to low melatonin under low salt stress in soil C (Table 4).

**Table 4:** Responses of bacterial and fungal communities to individual treatments of melatonin under various stressor conditions as determined by PERMANOVA.

		Cadmium stress									Salt stress								
		Soil P			Soil C			Soil W			Soil P			Soil C			Soil W		
		H	L	Z	H	L	Z	H	L	Z	H	L	Z	H	L	Z	H	L	Z
Bacteria	High MT vs. Low MT																		
	High MT vs. No MT																		
	Low MT vs. No MT																		
Fungi	High MT vs. Low MT																		
	High MT vs. No MT																		
	Low MT vs. No MT																		

*For control treatments, melatonin was replaced with dilute ethanol. Significant differences (*p <* 0.05) between melatonin treatments are highlighted under the following significance levels: 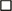: *p* > 0.05 (n.s.); 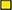: 0.01 < *p* ≤ 0.05; 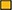: 0.001 < *p* ≤ 0.01; 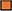: *p* ≤ 0.001. MT, melatonin; H, high stressor; L, low stressor; Z, no stressor. All treatments and controls for the same soil were composed of a standardized amount of dilute ethanol. *n* = 4 replicates per treatment. Statistical values presented in Supplementary Table S6*.

### Microbial Responses Within Soil W Communities

Only soil W under salt stress showed significant (*p <* 0.01) responses to treatments of melatonin, stressor, and melatonin × stressor for both fungal and bacterial communities as described by PERMANOVA (Table 3, Supplementary Tables S1, S5, S6). These communities were subsequently assessed in greater detail.

#### Bacterial Communities Show Distinct Responses to Melatonin Under Salt Stress (Soil W)

The nMDS plot for bacterial assemblages showed clear separation of samples according to treatments, with both melatonin and salt treatments resulting in shifts in community structures compared to the control (Figure 2A). Various individual OTUs shifted in response to the different treatments (Figure 2B, Supplementary Figure S7A). Bacterial communities treated with melatonin were substantially different [PERMANOVA *F*_(2, 27)_ = 7.839, *p <* 0.001] to control communities, with high melatonin concentration being 50.46% dissimilar to the control treatments whereas low melatonin concentration samples showed 48.05% dissimilarity (Supplementary Table S7A). Similarity percentage analysis (SIMPER) showed the OTUs contributing most to assemblage differences upon high melatonin treatment in comparison to control samples. This analysis found that the top three OTUs accounted for 27% of the total dissimilarity. These were OTU: 849 bp (12.88%), 741 bp (7.32%), and 756 bp (6.83%). High and low concentration melatonin samples were 36.70% dissimilar to each other with 29 OTUs accounting for 71.05% of these differences, the highest of which (OTU: 180 bp) represented only 5.01% of the dissimilarity (Supplementary Figure S7A). Bacterial responses to both concentrations of melatonin for soil W were significant under high and low salt stress in comparison to the respective salt only treatments (Table 4). Under all the above comparisons, a single OTU (756 bp) consistently increased (up to 7.67%) when melatonin was present, independent of melatonin and salt concentrations. Interestingly, some OTUs most responsive to melatonin under high salt conditions in soil W (e.g., OTU 387 bp) were far less responsive to melatonin under low salt conditions and vice versa (e.g., OTU 849 bp).

#### Fungal Community Responses to Treatments (Melatonin and/or Salt) in Soil W

NMDS ordination for fungal communities within soil W indicated distinct separation of samples treated with melatonin-only [PERMANOVA: *F*_(2, 27)_ = 2.131, *p <* 0.001] and salt-only [PERMANOVA: *F*_(2, 27)_ = 6.027, *p <* 0.001] when compared with respective control samples (Figures 3A,B). Relative abundances of individual fungal OTUs varied across all treatments in soil W under salt stress (Supplementary Figure S7B). Fungal communities in soil W treated with high melatonin concentration showed greater dissimilarity (33.72%) than low melatonin-treated samples (28.54%) when both were compared with control samples (Supplementary Table S7B), with high melatonin treatment noticeably different (*p <* 0.05) to control samples (Table 4). In soil W communities treated with high melatonin concentration, three taxa accounted for almost half of the total dissimilarity: OTUs 617 bp (20.05%), 613 bp (14.05%), and 597 bp (9.37%). Correspondingly, OTUs 597 bp (13.63%) and 581 bp (9.23%) also accounted strongly for the community dissimilarity between control and low melatonin-treated samples (Supplementary Figure S7B). The separation between samples treated with low melatonin concentration only compared with samples treated with both salt and low melatonin concentration was observed; however, fungal community differences were not determined as significant in response to melatonin under salt stress (Figure 3A, Table 4).

## Discussion

Soil microbial communities are a key component of a healthy ecosystem, providing a number of ecosystem services including direct and indirect nourishments of plant root systems (Wall and Virginia, 1999; Yao et al., 2000; Kirk et al., 2004; Wahid et al., 2016). We detected significant responses of microbes at a community level to melatonin treatment under normal or abiotic stress conditions in agricultural top-soils (10 cm depth). These responses varied slightly according to the melatonin dose and differed considerably between bacteria and fungi. As shifts in soil microbial communities may result in changes to various ecosystem services provided by soil microbes (Mau et al., 2019; Zhang et al., 2019), understanding how soil microbes respond to melatonin may be an important aspect in determining the viability of potential future agricultural applications of melatonin.

The doses of melatonin chosen in the current study (4 and 0.2 mg kg^−1^ soil) correspond to equivalent concentrations of approximately 100 and 5 μM, respectively. Enhanced abiotic stress tolerance as well as bio-stimulation have been reported in plants treated with exogenous melatonin within relative close proximity to either of these concentrations (Zhang et al., 2015; Nawaz et al., 2016; Sharif et al., 2018; Wang et al., 2018; Debnath et al., 2019). Therefore, these doses have biological relevance related to future melatonin applications in agriculture. Foliar spray of 100 μM melatonin on wheat (*Triticum aestivum* L. cv. Pandas) resulted in enhanced plant growth and reduced oxidative damage caused by exposure to cadmium (Kaya et al., 2019). Similarly, tolerance to salinity stress was increased in *Malus huphensis* upon pre-treatment with 100 μM melatonin (Li et al., 2012). Various studies also present the beneficial effect of low melatonin treatments (≤10 μM) for plants. For example, pre-treatment of oxidatively stressed *Arabidopsis* with 5 μM melatonin resulted in strong upregulation of genes involved in autophagy (Wang et al., 2015). Seed germination and seed imbibition were improved by 1 μM melatonin under salt stress conditions (Zhang et al., 2017a,b).

We hypothesized that melatonin would alter soil microbial community structures, as previous reports suggest that melatonin may act with antimicrobial properties. For example, melatonin was shown to inhibit the *in vitro* growth of the human bacterial pathogens, *Staphylococcus aureus, Pseudomonas aeruginosa*, and *Acinetobacter baumannii* at concentrations between 130 and 530 μM (Tekbas et al., 2008). Melatonin has also been reported to inhibit the *in vitro* growth of the human pathogenic yeast, *Candida albicans*, albeit at a much higher concentration (1,300 μM) (Öztürk et al., 2000). Our community study complements these findings, as bacteria associated with the three different agricultural soils were affected by melatonin alone. We also hypothesized that fungal communities would be less responsive to melatonin compared with bacteria, as previous *in vitro* studies have indicated limited responses to melatonin by fungi (Table 2, Supplementary Table S6). For example, *in vitro* studies investigating responses of filamentous fungi to melatonin found that at very high concentration (100 mM), melatonin showed no impact on the *in vitro* growth of *Physalospora piricola, Botrytis cinerea* or *Mycosphaerella arachidicola* (Wang et al., 2001). Growth of *Alternaria* spp. has been reported to be inhibited at a relatively high melatonin concentration (4 mM) (Arnao and Hernández-Ruiz, 2015).

Fungal communities responded far less to exogenous melatonin compared to bacteria in our studies (Table 3). One important factor influencing the limited responses of fungal communities to melatonin may have been the duration of incubation. In our current study, the 10-day incubation period was selected to accommodate both bacterial and fungal responses to treatments. Microbial activity is generally considered to be stabilized 7–10 days post-treatment exposure (Chowdhury et al., 2011). However, a longer incubation duration may have allowed a greater fungal community response as slower growing fungi would have had more time to sufficiently respond to melatonin treatments.

Both melatonin doses resulted in distinct shifts of bacterial and fungal communities, with high melatonin treatments resulting in slightly greater shift compared to low doses (Table 4). Melatonin may be utilized more efficiently by some microbes compared to others under the various experimental conditions, thus, potentially offering a competitive advantage to select groups. Our study indicated that some microbes accounted strongly for the shifts in communities treated with melatonin across the three soils (Supplementary Tables S3, S4; Supplementary Figure S7). Some root-dwelling bacteria have been shown to secrete melatonin (Jiao et al., 2016). The plant hormone Indole-3-acteic acid (IAA) is structurally similar to melatonin (Arnao and Hernandez-Ruiz, 2014) and has been shown to act as a signaling molecule between soil bacteria, fungi, and plant roots, resulting in a complex dynamic of responses by individual microbes within the rhizosphere community (Lambrecht et al., 2000; Lee and Lee, 2010; Spaepen and Vanderleyden, 2011; Fu et al., 2015). Therefore, investigating the possibility that melatonin may also be utilized in the rhizosphere to influence microbial community dynamics is worthy of further research.

Recently, Li et al. (2018) investigated the effect of exogenous melatonin application (200 μM; applied at 20-day intervals for 6 months), without abiotic stress, in two soils types associated with horticultural practices (apple orchard and vegetables) by sampling the subsoil region (20–30 cm depth). Bacterial compositions of melatonin-treated soil samples were shown to be similar to controls; however, some genera, many unknown, shifted strongly in response to melatonin (Li et al., 2018). In fungal assemblages, *Ascomycota*, in particular, were negatively affected by melatonin, resulting in a greater establishment of *Glomeromycota* and *Basidiomycota* (Li et al., 2018). While a direct comparison between our study and this report is difficult, these results are analogous to the trends observed in our investigation and indicative of the importance of soil agricultural histories in microbial response to melatonin. In our study, we used topsoils, which are generally more microbially rich (compared to subsoils), as well as soils associated with agricultural practices (crop production and pasture). Further analysis by taxa identification of the soil microbial communities associated with responses to melatonin would determine those microbes associated with particular ecological niches (e.g., mycorrhizal fungi or plant growth-promoting rhizobacteria) and if they are known to be beneficial to crops. Based on our findings, we recommend that plant-based studies applying melatonin through the soil should also take into consideration the effect treatments may have on soil microbes as part of their investigations.

The responses by bacteria to melatonin under unstressed and stressed conditions were not consistent across the three soils (Table 4), indicating that numerous factors may determine the interactions of soil bacteria with melatonin. These different responses may have been in part due to differing soil physiochemical characteristics between the soils, as well as differing interactive effects of treatments with various soil characteristics (Zhong and Cai, 2007; Ahn et al., 2012; Zhao et al., 2014; Geisseler et al., 2017). In the current study, some communities were less impacted by abiotic stress upon the availability of exogenous melatonin. However, as this trend was not observed under cadmium stress, it may be possible that melatonin was utilized by soil bacteria to sustain natural microbial activity by coping with impacts specific to salt stress, such as enhanced osmotic pressure and ion toxicity (Morrissey et al., 2014; Yan et al., 2015).

Melatonin has been consistently shown to reduce cellular levels of ROS in plants exposed to abiotic stress (Tan et al., 2012), by either acting as a highly efficient antioxidant (Reiter et al., 2015, 2016), or as a signaling molecule, resulting in the upregulation of gene expression, or increased enzyme activities of ROS-scavenging enzymes (Rodriguez et al., 2004; Lee et al., 2014; Manchester et al., 2015; Zhang et al., 2015; Nawaz et al., 2018). Under abiotic stresses, such as cadmium and salt, plants can cope better by adjusting physiological and enzymatic processes when melatonin is applied (Li et al., 2012, 2016; Byeon et al., 2015; Liang et al., 2015; Jiang et al., 2016; Gu et al., 2017). Some reports also suggested that melatonin may enhance abiotic stress tolerance in microbes as endogenous levels of melatonin increased in *Trichoderma* spp. (Liu et al., 2016) and *Saccharomyces cerevisiae* (Rodriguez-Naranjo et al., 2012) under abiotic stresses (cadmium and ethanol respectively).

Plant growth and development are greatly impacted on soils contaminated with cadmium or salt, resulting in reduced crop yield and food quality (Qadir et al., 2014; Shao et al., 2019; Wang et al., 2019). Cadmium chloride hemipentahydrate (CdCl_2_.2½H_2_O; 280 or 100 mg kg^−1^ soil) and salt (NaCl; 7 or 4 g kg^−1^ soil) were separately applied as abiotic stressors in the current study. These stressor concentrations were selected to be within the range of those reported to induce effects on soil microbial activities in previous studies for cadmium (Cáliz et al., 2013; Wood et al., 2016a,b) and salt (Rath et al., 2016). For example, Wood et al. (2016a,b) found that soil treated with cadmium chloride (CdCl_2_) at 100 mg kg^−1^ soil resulted in significant shifts in bacterial community structures, whereas fungal communities were unaffected at this concentration of soil contamination. Cáliz et al. (2013) found that significant impacts to microbial communities in soils are treated with cadmium sulphate (CdSO_4_) at 1,000 mg kg^−1^ soil. Salinity altered the bacterial and fungal community structures of root microsymbionts associated with alder (*Alnus glutinosa*) (Thiem et al., 2018). Rath et al. (2016) found effects on soil bacterial and fungal growth as well as microbial respiration at 63, 55, and 79 mmol NaCl kg^−1^ soil, respectively. This corresponds to similar concentrations of NaCl applied in the current experiment for 4 g kg^−1^ (70–96 mmol NaCl kg^−1^ soil). Our results indicate that the stressor concentrations affected bacterial and fungal community structures in all three soils (Table 3). Previous soil studies have found soil bacterial communities to be more responsive to various stress treatments in comparison to fungal communities, with fungi showing greater tolerance to abiotic stressors (Hiroki, 1992; Müller et al., 2001; Marschner et al., 2003; Rajapaksha et al., 2004). Interestingly, we found that bacterial communities showed more distinct responses to melatonin under abiotic stress conditions compared to fungi, along with more distinct separation of communities per treatment (e.g., Soil W). This may potentially suggest that bacteria utilized melatonin better than fungi under abiotic stress conditions.

As melatonin is safe for human consumption and can be applied to plants in numerous ways such as seed coating, foliar spraying or soil treatment, it may, therefore, have a major role in future agricultural practices for crop yield protection and improvement (Janas and Posmyk, 2013; Wei et al., 2015; Cui et al., 2017; Zhang et al., 2017a,b). Higher amounts of melatonin in foods due to exogenous application on crops and vegetables have a potential beneficial effect on human health, especially if it results in a reduced dependency on pesticide practices in agriculture. However, the chemically synthetized melatonin powder used in this study (>98% purity) costs AU$146 per gram (Sigma Aldrich Pty. Ltd., Australia), and at that price, it would not be cost-effective for use in agriculture, even with a highly efficient application such as seed coating for example. Although some other “melatonin powder” can be purchased online and at cheaper cost there is few to no information about their provenance and quality. Further research is required to ensure the efficacy of lower grade melatonin for agricultural use matches that of the pure powder form. A recent study reported the production of melatonin by yeast (*S. cerevisiae*) using a glucose-based approach (Germann et al., 2016). This potentially offers a far more sustainable and economical method of producing melatonin. Developing an affordable, large-scale method of producing melatonin is a vital logistical aspect regarding the future use of melatonin in agricultural practices.

## Conclusion

In conclusion, this study has demonstrated that exogenous melatonin altered the structures of soil bacterial and, to a lesser extent, fungal assemblages under unstressed and abiotic stressed conditions. No previous reports have examined the effects of melatonin on agricultural soil microbial communities under abiotic stress. Some bacterial and fungal OTUs responded strongly (positively or negatively) to melatonin, indicating that melatonin may alter the growth of specific groups of microbes. At a community level, responses to treatments varied across the three soils, indicating that the interaction between soil microbes with melatonin is a complex process, influenced by numerous factors including physiochemical characteristics and differing community compositions across soils. Further research is required to profile the microbial taxa responsive to melatonin as well as investigate potential functional associations between melatonin with abiotic stress tolerance in microbes. The main factors (e.g., soil chemistry) causing the differences in natural microbial communities between the different soils also requires further analysis. Additional research is also required to determine if specific soil characteristics influence the responses of microbial communities to melatonin. Moreover, studies may explore potential plant-microbe interactions in soil upon the bioavailability of exogenous melatonin. Future studies involving ameliorating plant stress using melatonin should take into account the potential impact of soil microbiota and the subsequent impact on plant-microbe interactions (beneficial as well as pathogenic). Understanding the role of melatonin in soil microbial community dynamics may provide vital information regarding the viability of melatonin application relating to future agricultural practices.

## Data Availability Statement

All datasets generated for this study are included in the article/Supplementary Material.

## Author Contributions

AM, FB, AF, and KP conceived and designed the study. AM performed the experiments and data analysis with the assistance of EE, and AM interpreted the results and wrote the manuscript with suggestions from FB and KP. All authors discussed the results and commented on the manuscript. AF and KP financed the research.

### Conflict of Interest

The authors declare that the research was conducted in the absence of any commercial or financial relationships that could be construed as a potential conflict of interest.
